# The percutaneous spinal endoscopy “isolation zone” technique for discogenic low back pain: a case series study

**DOI:** 10.1186/s40001-022-00837-2

**Published:** 2022-10-14

**Authors:** Lu Wang, Lingxia Li, Cai Cheng, Yuan Xue

**Affiliations:** 1 Department of Orthopedics, Cangzhou Central Hospital, Tianjin Medical University, Cangzhou, 061001 Hebei China; 2 Department of Clinical Pharmacy, People’s Hospital of Cangzhou City, Cangzhou, 060002 Hebei China; 3 Department of Orthopedics, Tianjin Medical University General Hospital, Tianjin, 300052 China

**Keywords:** Low back pain, Endoscopy, Nerve block, Treatment outcome, Case series

## Abstract

**Background:**

This study aimed to explore the clinical values of the percutaneous spinal endoscopy “isolation zone” technique for discogenic low back pain (DLBP).

**Methods:**

This retrospective case series study enrolled patients with intervertebral DLBP treated with the percutaneous spinal endoscopy “isolation zone” technique in the department of Orthopedics, Cangzhou central Hospital affiliated to TianJin Medical University between September 2017 and September 2020.

**Results:**

Forty-five patients with DLBP were enrolled. The mean operation time was 94.7 ± 17.7 min. The visual analogue scale (VAS) score of lumbosacral pain was 6.95 ± 1.02 before operation, 2.64 ± 0.71, 1.80 ± 0.54, 1.42 ± 0.50, and 1.27 ± 0.45 at 1, 3, 6, and 12 months after operation, respectively. The Oswestry disability index (ODI) score of low back pain was 72.84 ± 5.95 before operation, 35.1 ± 5.30, 25.22 ± 4.85, 16.78 ± 4.63, and 10.91 ± 2.36 at 1, 3, 6, and 12 months after operation, respectively. At final follow-up, the treatment effect based on modified MacNab criteria was excellent in 24 cases, good in 13 cases, and fair in 8 cases. The excellent/good rate was 82.2%.

**Conclusion:**

The percutaneous spinal endoscopic “isolation zone” technique seems to be a promising surgical alternative for DLBP.

## Background

Discogenic low back pain (DLBP) is a common type of spinal degenerative disease. DLBP results from multifactorial changes due to intervertebral disc degeneration that interact with the nervous system to induce pain [1]. The pain manifests with or without leg pain and is sometimes associated with sitting intolerance, an extension catch, difficulty lifting, or an inability to maintain the same posture [2]. The clinical symptoms of DLBP often occur repeatedly for a long time, causing great pain to patients, seriously affecting their quality of daily life and can become a serious medical and social problem responsible for disability both in work and recreation [3, 4].

The traditional treatment of DLBP is mainly conservative such as rest, drug treatment, or physiotherapy, but this often cannot fundamentally solve the problem. For example, in cases of an intervertebral disc annulus fibrosus tear [5]. For patients with ineffective conservative treatment, lumbar fusion surgery is often used to remove the degenerative intervertebral disc and stabilize the diseased segments [6]. However, traditional fusion surgery destroys the normal and stable structure of the spine, with high treatment cost and great surgical trauma, and the improvement of lumbar pain, spinal function, and quality of life after fusion is not satisfactory [7].

Minimally invasive methods have been developed to improve the surgical outcomes of lumber disc surgery, such as the Yeung endoscopic spine system (YESS) technique [8, 9], intradiscal radiofrequency therapy (PIRFT), and intradiscal electrothermal therapy (IDET) [10, 11]. The treatment principle is to treat the annulus fibrosus fissure through intradiscal radiofrequency or high temperature, so as to destroy the pathway formed by inflammatory mediators in pain transmission. However, due to the limitation of puncture location, the postoperative effect in some patients is poor [12]. Our department applied the "isolation zone" technique of spinal endoscopy to treat the patients with DLBP in the spinal canal and intervertebral disc. The “isolation zone” technique aims to block the main sensory nerve—the sinuvertebral nerve, which is densely covered with pain conductors on the surface of the fibrous ring behind the intervertebral disc and the posterior longitudinal ligament. In addition, the pathogenic inflammatory medium in the spinal canal and the intervertebral disc is cleaned up [13]. The technique may be more effective than previous methods.

Therefore, this study aimed to evaluate the clinical values of the percutaneous spinal endoscopy “isolation zone” technique for DLBP.

## Methods

### Study design and population

This case series study enrolled patients with DLBP treated by spinal surgery in the department of Orthopedics, Cangzhou central Hospital affiliated to TianJin medical University between September 2017 and September 2020. The Ethics Committee of the Cangzhou central Hospital affiliated to TianJin medical University approved this study (No. 20210205). All patients provided written informed consent.

**Fig. 1:**
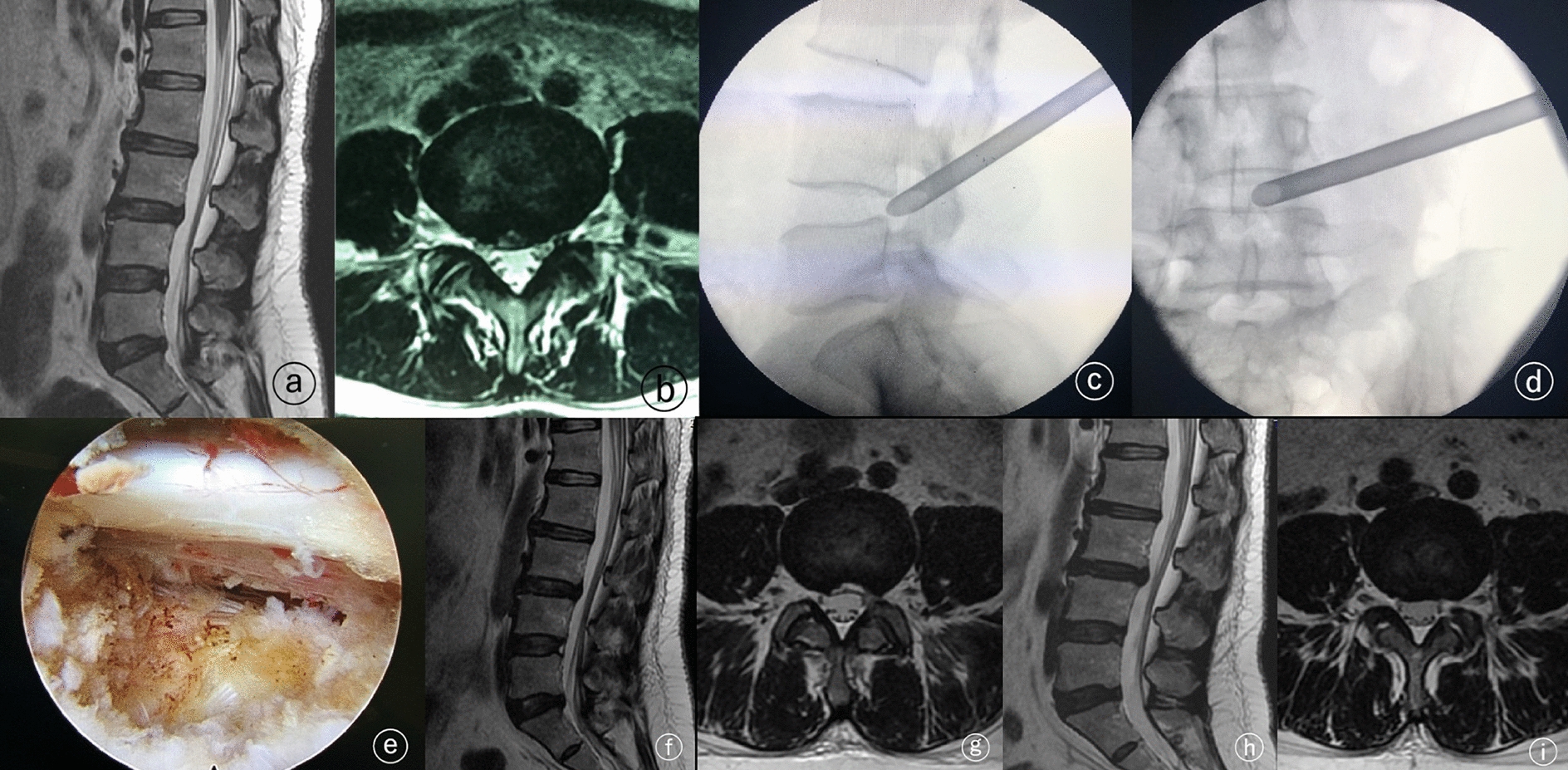
**A**, **B** High-intensity zone of L4/5 segment intervertebral disc annulus fibrosus can be seen in the preoperative lumbar MRI; **C**, **D** the working channel of spinal endoscopy during operation; **E** intraoperative images showed the “isolation zone” formed in the nerve root and ventral dura after endoscopic decompression; **F**, **G** one week after operation, the lumbar MRI was rechecked, and the signal of fibrillar ring tear disappeared; the range of ventral decompression of dura mater was sufficient; **H**, **I** the lumbar MRI was reexamined 10 months after operation, the tear of fibrous ring disappeared; the surgical scope of the "isolation zone" healed well

The inclusion criteria were as follows: (1) patients with low back and lumbosacral pain symptoms, without typical sciatic nerve pain. Prolonged sitting, standing, bending, and physical labor could cause pain and discomfort in the lumbar back, hip, greater trochanter, and groin, and it was generally a dull pain; (2) preoperative X-ray, computed tomography (CT), magnetic resonance imaging (MRI) and/or other imaging data showed single segment disc lesions, mostly mild herniation of the intervertebral disc, no stenosis of bilateral lateral recess, and no obvious collapse of intervertebral space; (3) there was a high signal area of annulus fibrosus tear behind the responsible segment disc, and the MRI image showed a hiz (high intensity zone) (Fig. 1 A, B); (4) physical examination showed no serious lower limb nerve dysfunction, and straight leg rising test was negative; (5) conservative treatment (including rest, oral medicine, acupuncture, physiotherapy, muscle function exercise) had been tried at least 3 months and was ineffective, and the symptoms seriously affected daily life and work; (6) using a mixture of low concentration lidocaine and triamcinolone acetonide for intervertebral foramen responsible segmental nerve block, the visual analogue scale (VAS) of pain decreased by more than 60% within 24 h. The exclusion criteria were: (1) patients with local segmental spinal instability or slippage; (2) patients complicated with spinal tumor or myelopathy; (3) patients with coagulation dysfunction; (4) patients complicated with mental disorders; (5) patients with incomplete data.

### Data collection

Clinical data were collected from the clinical records, including age, sex, operation time, and spinal levels. The VAS score and Oswestry disability index (ODI) score of low back pain and pain around the buttocks were measured before operation and 1, 3, 6, and 12 months after operation. The treatment effect was evaluated by modified MacNab criteria at the last follow-up, alongside lumbar MRI imaging, and reporting of any complications.

The modified MacNab criteria [14] evaluated the overall treatment effect of surgery, using four grades: Excellent: no pain, no restriction of mobility, return to normal work and level of activity; Good: occasional non-radicular pain, relief of presenting symptoms, able to return to modified work; Fair: Some improved functional capacity, but still handicapped by intermittent pain. Poor: No relief of symptoms. The VAS evaluated the degree of pain using a ruler that provides a range of scores from 0 to 10, where 0 means no pain and 10 represents unbearable pain. A higher score indicates greater pain intensity. The ODI is a measure to evaluate spinal disorders and patient progress in clinical practice. Scores of 0–20% are considered mild dysfunction, 21–40% are considered moderate dysfunction, 41–60% are considered severe dysfunction, and 61–80% are considered disability. Cases with scores of 81–100% are either long-term bedridden or exaggerating the impact of pain on their life.

### Typical surgery procedures

All patients were operated by a single senior physician skilled in spinal endoscopy in our research center. Regular oral nonsteroidal anti-inflammatory analgesics (such as diclofenac sodium) and central skeletal muscle relaxants (such as tizanidine hydrochloride) were administered 48 h before operation. All patients underwent the operation in the lateral position under the guidance of a G arm X-ray machine.

Local anesthesia (1% ropivacaine 10 ml 2% lidocaine 30 ml 0.9% saline 45 ml) was used in all patients. The anesthesia levels were skin and subcutaneous, lumbar dorsal fascia, articular process joint, and disc surface.

All enrolled patients were treated with the “isolation zone” technique under percutaneous spinal endoscopy. The specific method involved puncture of the tip of the articular process on the target intervertebral space with an 18G puncture needle. The tip of the needle was located on the posterior edge of the intervertebral joint, and the anterior edge of the intervertebral joint. The guide wire was placed, the skin was cut about 7 mm, to place 2, 3.5, 4.5, and 5.5 mm catheters step by step to expand the soft tissue. The Tom Shidi needle was then placed along the guide wire, and the tip of the Tom Shidi needle was fixed at the tip of the facet joint under fluoroscopy, and pointed to the protruding target of the intervertebral disc. This was then used to gently hammer the bone penetrating the facet joint, while appropriately adjusting the depth of the Tom Shidi needle into the spinal canal according to the protruding position of the intervertebral disc. The guide wire was replaced after the positioning was confirmed by frontal and lateral fluoroscopy. Bone drills with diameters of 4.5, 6, 7, 8, and 9 mm were successively inserted along the guide wire for intervertebral foramen plasty. The guide wire was replaced again and the expansion guide rod was inserted and the 7.5-mm working channel was inserted along the guide rod. It was confirmed that the working channel reached the target through frontal and lateral fluoroscopy (Fig. 1C, D). The spinal endoscope was then placed through the working channel. Nucleus pulposus forceps were used, through the central working channel of the 3.7-mm endoscope, to clean the local soft tissue and explore the annulus fibrosus tear. The red inflammatory soft tissue scattered on the surface and the nucleus pulposus tissue with severe degeneration and poor elasticity below were mainly cleaned, and the annulus fibrosus tear was removed with forceps. Flexible bipolar radiofrequency was used to denervate the edge of the fibrous ring and the interior of the intervertebral nucleus pulposus. Forceps were used to moderately remove some fibrous rings of the intervertebral disc to the proximal, caudal, and contralateral sides. The removal range was that the proximal side could reach the posterior lower edge of the upper vertebral body, the caudal side could reach the posterior upper edge of the lower vertebral body, and the longitudinal fibers of the posterior longitudinal ligament could be exposed on the contralateral side. When there was sufficient space on the ventral side of the nerve root and the dural sac during the operation of the responsible segment, the edge and surface of the residual fibrous ring and the surface of the affected side of the posterior longitudinal ligament was electro coagulated and denervated by flexible bipolar radiofrequency. When the blood vessels on the surface of the nerve root were filled, there was autonomous pulsation, and the ventral and dorsal space was sufficient (Fig. 1E), after the patient’s subjective symptoms were reduced, the endoscope and working channel was withdrawn, and the incision was sutured.

Patients received routine administration of infection prevention, dehydration and detumescence, neurotrophic and corresponding symptomatic treatment after operation. On the first day after the operation, patients used waist orthosis or support to get out of bed. The waist orthosis or support continued for 3 weeks, to allow the patients to get out of bed step by step. The patients were guided to do lumbar dorsal muscle function exercise, straight leg raising (SLR) exercise, and a lumbar spine health care program daily.

### Statistical analysis

The data were analyzed by SPSS 22.0 statistical software (IBM Corp., Armonk, NY, USA). Continuous variables with a normal distribution were expressed by mean ± standard deviation (SD), and categorical variables were expressed as numbers (percentages). Comparisons for continuous data were performed using Student’s *t*‐test or one‐way analysis of variance (ANOVA). Two-tailed *P*-values < 0.05 were considered significant.

## Results

A total of 45 patients with DLBP, with mean age 48.3 ± 10.1 (range 26–62) years old were included. 29 cases were L4/5, 16 cases were L5/S1. Among the 45 patients, there was no forced interruption due to intolerable pain of local anesthesia. The mean operation time was 94.7 ± 17.7 (range: 65–125) min. The VAS score of lumbosacral pain was 6.95 ± 1.02 before operation, 2.64 ± 0.71, 1.80 ± 0.54, 1.42 ± 0.50, and 1.27 ± 0.45 at 1, 3, 6, and 12 months after operation, respectively. The ODI score of low back pain was 72.84 ± 5.95 before operation, 35.1 ± 5.30, 25.22 ± 4.85, 16.78 ± 4.63, and 10.91 ± 2.36 at 1, 3, 6, and 12 months after operation, respectively. There was significant improvement compared with that before operation (all *P* < 0.05). At the last follow-up, the treatment effect according to the modified MacNab criteria was excellent in 24 cases, good in 13 cases, and fair in 8 cases. The excellent and good rate was 82.2% (Table 1).

**Table 1:** Baseline characteristics of patients with DLBP

	Patients (*n* = 45)
Age, years	48.3 ± 10.1 (range 26–62)
Male	
Spinal levels	
L4/5	29 (64.4)
L5/S1	16 (35.6)
Operation time, min	94.7 ± 17.7 (65–125)
VAS score	
Pre-operation	6.95 ± 1.02
1 month after operation	2.64 ± 0.71
3 months after operation	1.80 ± 0.54
6 months after operation	1.42 ± 0.50
12 months after operation	1.27 ± 0.45
ODI	
Pre-operation	72.84 ± 5.95
1 months after operation	35.1 ± 5.30
3 months after operation	25.22 ± 4.85
6 months after operation	16.78 ± 4.63
12 months after operation	10.91 ± 2.36
Treatment effect	
Excellent	24 (53.3)
Good	13 (28.9)
Fair	8 (17.8)

Postoperative reexamination of lumbar MRI showed that the tears of the fibrous ring at the responsible segment disappeared in all patients, and the “isolation zone” at the ventral side of the dura mater and the ventral side of the nerve root healed well (Fig. 1F–I).

One case of postoperative femoral nerve paralysis was treated conservatively with neuronutrition, acupuncture physiotherapy and functional exercise, and the symptoms disappeared 4 weeks after operation. One had neck and back pain during the operation, which was considered as spinal cord like hyperbaric reaction. The symptoms disappeared 30 min after the removal of water pressure and oxygen inhalation. There were no serious complications such as permanent nerve injury and intervertebral space infection.

## Discussion

The results show that VAS score of lumbosacral pain and ODI score of low back pain improved after the operation. At final follow-up, the treatment effect was excellent in 24 cases, good in 13 cases, and fair in 8 cases. The excellent/good rate was 82.2%. The results suggest that the percutaneous spinal endoscopic “isolation zone” technique may be a promising surgical alternative for DLBP.

Minimally invasive methods have been used to treat patients with DLBP if conservative treatment fails to improve symptoms. The results of this study showed that pain as measured by VAS score dropped from 6.95 ± 1.02 before operation to 1.27 ± 0.45 at 12 months after operation in the patients treated with the “isolation zone” technique. This compares well to recent studies that have used different methods. For example, one study used transsacral epiduroscopic laser decompression (SELD) in 52 patients [15]. In that study, the VAS score fell from 5.6 to 1.2 at 12 months [15]. Another study that used a transforaminal endoscopic system (TESSYS) in 62 selected patients found VAS decreased from 6.7 ± 2.5 to 1.0 ± 0.6 at last follow-up [16]. Percutaneous endoscopic treatment for annular tear in selected patients with DLBP was applied in 24 patients by using the outside-in technique [17]. At 12 months VAS fell to 1.62 ± 0.77 from the preoperative value of 6.83 ± 0.87 [17]. So, pain improved significantly using all of these methods. However, the ability to compare the studies is limited to some degree by differences in the studies, such as different patient populations.

The endoscopic methods above also improved spinal function as indicated by ODI. In this study, the ODI decreased from 72.84 ± 5.95 preoperatively to 10.91 ± 2.36 at 12 months postoperatively. The studies described above also showed the ODI score dropped from the preoperative level of 22.3 to 8.8 at 12 months [15], 35.8 ± 5.4 to 8.7 ± 2.1 at last follow-up [16], and 61.58 ± 5.37 to 12.26 ± 1.76 at 12 months [17]. When the treatment effect was evaluated, the success rate in this study was 82.2% at 12 months. This also compares well with other studies showing success rates of 75.8% [16] and 91.7% [17].

DLBP is one of the common spinal degenerative diseases in clinic. It mainly manifests as atypical low back pain and lower limb pain. The pain is located mostly in the lower waist, hip and groin areas, posterolateral thigh, and knee joints. The positioning is inaccurate. The symptoms worsen when standing, sitting, or bending for long periods [4, 18]. There is no typical sciatica, and the straight leg elevation test is mostly negative [19]. At present, the pathogenesis of DLBP is thought to mainly involve local rupture of annulus fibrosus caused by intervertebral disc degeneration, and the production of inflammatory factors that stimulate the pain receptors of sinus and vertebral nerves densely covered with intervertebral discs to cause pain [20]. The nerve endings proliferating in the nucleus pulposus at the annulus fibrosus gap induce lumbar pain under the combined action of nucleus pulposus pressure stimulation and inflammatory mediators [21]. The central type of intervertebral disc herniation and fibrous ring tear squeeze the posterior longitudinal ligament and dural sac backward to form continuous stimulation of inflammatory media and form synergistic lumbar pain. Due to stimulation of inflammatory mediators, a large number of neovascularized pannus are formed in the area around the annulus fissuring, gradually forming scattered inflammatory lesions, aggravating the pain stimulation of new nerve fibers [22]. In this study, the surgeon performed the clean-up of the annulus fibrosus rupture area and removed ruptured annulus fibrosus under spinal endoscopy, and the follow-up MRI after surgery showed that the hiz signal of the mild bulging disc area before surgery had partially or totally disappeared, and the lumbosacral pain triggered by the lumbar exercise had been largely alleviated, we believe that endoscopic manipulation of the annulus fibrosus rupture area blocking the production source and the conduction pathway of inflammatory factors. It is significant for the resolution of discogenic low back pain.

For DLBP, it is very necessary to determine the responsible segment. In addition to the lumbar MRI showing signal changes of the intervertebral disc with hiz and modic signs and Schmorl nodules on the posterolateral side, discography, induction test, and transforaminal nerve block are important diagnostic techniques [23]. The angiography and induction test injects methylene blue into the intervertebral disc of the responsible segment [24]. During intervertebral disc imaging, methylene blue contrast agent flows out from the nucleus pulposus to the outer layer of the annulus fibrosus through the inner layer gap of the annulus fibrosus. The pressure generated by the contrast agent acts on the granulation tissue and the nerve fibers distributed therein, inducing the aggravation of lumbar pain, which is the basis for the replication of lumbar pain [25]. However, methylene blue is destructive to the normal intervertebral disc tissue [26]. In addition, a false-positive induction test will also lead to inaccurate preoperative judgment of responsible segments. Sometimes, it is necessary to make comparison of adjacent normal segments, which also increases the complexity of preoperative diagnosis [27]. Therefore, we prefer to use transforaminal nerve drug block for preoperative diagnostic treatment. Under local infiltration anesthesia, the mixture of low concentration lidocaine and triamcinolone acetonide is injected into the lateral surface of the diseased intervertebral disc, and the analgesic effect is achieved by blocking the continuous nerve activity that produces pain. Transforaminal nerve root block has high diagnostic value in DLBP. By confirming the responsible segment and observing the symptom relief, a clear diagnosis can be made and a reference could be established for the follow-up endoscopic treatment. Patients with DLBP can undergo segmental block surgery if their VAS decreases by ≥ 60% within 24 h after nerve root block via intervertebral foramen, otherwise it may be necessary to increase endoscopic decompression segments or change the treatment plan.

Although there are many treatment schemes for DLBP, such as lumbar fusion surgery, intradiscal intervention technologies such as intradiscal electrotherapy, intradiscal injection of platelet rich plasma or hepatocytes, or intradiscal ozone technology the above technologies are controversial at present, and the treatment results are often uncertain [28, 29]. Compared with the traditional spinal endoscopic nucleus pulposus removal, the details of this “isolation zone” technology in the treatment of DLBP are more complex. The key technical points of the “isolation zone” technique were cleaning the inflammatory hyperplasia tissue on the surface of intervertebral disc and nerve root, sinus vertebral plexus block, cleaning and denervation of annulus fibrosus tear, removal of protruding nucleus pulposus tissue and intervertebral disc formation, denervation around the posterior longitudinal ligament, and forming an “isolation zone” of inflammatory factors and pain nerve conduction around nerve root and dura mater. Therefore, all the pain conducting nerve sinus vertebral nerve distribution areas, including the inflammatory tissue around the nerve root, the fibrous ring and the surface of the posterior longitudinal ligament, the intervertebral disc are explored, including the whole running area of nerve root canal and the inflammatory tissue around the outlet nerve root.

Our experience suggests that the “isolation zone” spinal endoscopy technology used in this study has the following advantages in the treatment of DLBP: (1) nerve block through the intervertebral foramen can accurately determine the responsible segment of DLBP, and avoid the damage to the intervertebral disc caused by the traditional intradiscal injection of contrast agent and the false-positive test; (2) endoscopic treatment of the responsible segment through the intervertebral foramen not only has less damage to the stable structure of the lumbar spine, but also preserves the spinal motor unit. It has advantages over the traditional fusion surgery in preventing the adjacent segment lesions after lumbar surgery; (3) under local anesthesia, the patients can autonomously reflect the nerve function of the lower limbs during the operation. The risk of nerve injury is small, and the postoperative recovery is fast. Patients can exercise early; (4) the pathogenic factors of the responsible segments are comprehensively treated to achieve the purpose of treating DLBP.

There were several limitations. Firstly, this was a single center study, and the sample size is limited. As a retrospective analysis there may have been some bias in the patients selection. There was no comparison group, so no clear conclusions can be made on the effectiveness of the treatment compared to standard treatment.

## Conclusion

The percutaneous spinal endoscopic “isolation zone” technique provided satisfactory clinical results for DLBP. The percutaneous spinal endoscopic “isolation zone” technique seems to be a promising surgical alternative for DLBP.

## Data Availability

The data and code used to support the findings of this study are available from the corresponding author upon reasonable request.
